# Prolonged Grief in Refugees Seeking Treatment for PTSD: Comorbidity with Post‐Traumatic Stress Symptoms and Network Structure

**DOI:** 10.1002/cpp.70097

**Published:** 2025-06-21

**Authors:** Franziska Lechner‐Meichsner, Mirjam Sophie Rueger, Kai Jannik Nehler, Thomas Ehring, Hannah Preiss, Nexhmedin Morina, Dana Churbaji, Ricarda Mewes, Julia Giesebrecht, Cornelia Weise, Regina Steil

**Affiliations:** ^1^ Department of Psychology University of Wuppertal Wuppertal Germany; ^2^ Department of Psychology Utrecht University Utrecht The Netherlands; ^3^ Department of Psychology Goethe University Frankfurt Frankfurt Germany; ^4^ Department of Psychology LMU Munich Munich Germany; ^5^ Institute of Psychology University of Münster Münster Germany; ^6^ Outpatient Unit for Research, Teaching and Practice, Faculty of Psychology University of Vienna Vienna Austria; ^7^ Department of Psychology Philipps‐University of Marburg Marburg Germany; ^8^ Clinical Psychology and Behavioral Health Technology, Department of Psychology Friedrich‐Alexander‐Universität Erlangen‐Nürnberg Erlangen Germany

**Keywords:** bereavement, complex, network analysis, post‐traumatic stress, prolonged grief disorder, PTSD, refugees, trauma

## Abstract

Refugees often experience traumatic events and the loss of loved ones, leading to post‐traumatic stress disorder (PTSD), complex PTSD (cPTSD) and prolonged grief disorder (PGD). It has been repeatedly shown that comorbidity between PGD and PTSD is frequent especially after violent losses, but only few studies have investigated the relationship between PGD and cPTSD. The network approach to psychopathology is well suited to investigate associations between different symptoms. We therefore combined a traditional investigation of the probable prevalence of PGD and its comorbidity with PTSD and cPTSD in refugees and used network analysis to identify central symptoms and bridge symptoms. A total *N* of 92 treatment‐seeking refugees who had experienced both loss and traumatic events completed a self‐report measure of PGD and clinical interviews for PTSD and cPTSD. We determined the probable prevalence of PGD_ICD‐11_ and rates of comorbidity. Network centrality and associations between symptoms of PGD and cPTSD were examined using network analysis. The probable prevalence of PGD_ICD‐11_ was 28.04%. Of those with probable PGD_ICD‐11_, 65.38% also met criteria for comorbid PTSD_ICD‐11_ and 19.23% for comorbid cPTSD. The most central PGD symptom in the network was *difficulties engaging in social or other activities*, and the most central cPTSD symptom was *negative self‐concept*. The most important PGD bridge symptom was *emotional numbness*. Results underscore the importance of screening for PGD in treatment‐seeking traumatized refugees in order to consider it in treatment planning. The relatively small sample size and the stability indices call for cautious interpretation of the results.

**Trial Registration:**

DRKS‐ID: DRKS00019876.

**Summary:**

- This is the first prolonged grief and post‐traumatic stress symptom network analysis in refugees.
- Prolonged grief disorder is an important mental health problem in refugees.
- Emotional numbness and avoidance were the most important bridge symptoms.
- Screening for PGD is important in traumatized treatment‐seeking refugees.
- Culturally sensitive grief‐focused interventions should be offered to refugees.

## 1 Introduction

The number of refugees resettling in Western countries continues to rise (United Nations High Commissioner for Refugees 2024), and many refugees have been exposed to potentially traumatic events (PTEs) related to war, persecution and flight (Nesterko et al. 2019; Nickerson et al. 2021). The prevalence of post‐traumatic stress disorder (PTSD) in refugees exceeds that in non‐refugee samples, with estimated pooled prevalences around 30% (Blackmore et al. 2020; Lechner‐Meichsner et al. 2024; Patanè et al. 2022). Moreover, refugees often experience repeated or chronic PTE, such as torture, that put them at risk for complex PTSD (cPTSD). Recently, cPTSD was added to the ICD‐11 as a sibling diagnosis to PTSD. It is characterized by three core PTSD symptom clusters (i.e., re‐experiencing, avoidance and a persistent sense of current threat) as well as disturbances in self‐organization (DSOs) that include affect dysregulation, negative self‐concept and difficulties in relationships (World Health Organization 2019). A recent systematic review reported an estimated cPTSD pooled prevalence of 57.4% in treatment‐seeking refugees (Lechner‐Meichsner et al. 2024).

Many refugees are also exposed to the loss of loved ones often under violent circumstances and alongside other PTE and migration‐related stressors. Refugees are therefore also at risk for prolonged grief (Djelantik, Smid, et al. 2020; Lechner‐Meichsner et al. 2024; Steil et al. 2019). Prolonged grief disorder (PGD) is a new diagnosis in ICD‐11 and DSM‐5‐TR that is characterized by persistent separation distress (American Psychiatric Association 2022; World Health Organization 2019). To warrant a diagnosis, core symptoms of yearning or longing and preoccupation with the deceased and accompanying symptoms such as sadness, guilt or difficulties accepting the death must persist for more than 6 months (ICD‐11) or 12 months (DSM‐5‐TR) beyond the death of the loved one. Reviews reported an estimated pooled prevalence of 33.2% for prolonged grief reactions (Kokou‐Kpolou et al. 2020) and estimated prevalences between 15.1% and 36.8% for PGD according to ICD‐11 (PGD_ICD‐11_) among bereaved refugees (Lechner‐Meichsner et al. 2024). Most of these estimates are, however, based on self‐report measurements, which can result in overestimation of prevalences (Stroebe et al. 2024).

PGD and PTSD share some similarities, yet they are distinct disorders (Boelen et al. 2010; Lenferink et al. 2021). Avoidance is present in both disorders; however, in PGD, it is typically directed at reminders of the loss, whereas in PTSD, it centres on internal and external reminders of the trauma. Intrusions are a core symptom of PTSD and can also occur in PGD. However, the primary emotion in PTSD is fear, whereas PGD is characterized by longing, yearning and a mix of positive and negative emotions. Both disorders can develop comorbidly after bereavement, especially after traumatic loss (e.g., Djelantik et al. 2017). A recent meta‐analysis showed that 49% of people with prolonged grief symptoms also have co‐occurring post‐traumatic stress symptoms (Komischke‐Konnerup et al. 2021). In addition, there is emerging evidence that PTSD and PGD are also often comorbid in refugees (Lechner‐Meichsner et al. 2024). Fewer studies have jointly investigated symptoms of PGD and cPTSD. Strong associations have been found between symptoms of PGD and symptoms of bereavement‐related cPTSD in people bereaved by suicide (Hofmann and Wagner 2024) and somewhat lower associations in refugees exposed to different traumatic events and the loss of a close person (Silove et al. 2017).

The importance of focusing on PGD in traumatized populations in general is strengthened by evidence that the presence of prolonged grief symptoms can exacerbate PTSD symptoms. Longitudinal studies have consistently shown that prolonged grief symptoms exert a greater influence on PTSD symptoms than the reverse (Janshen and Eisma 2024). During treatment, prolonged grief symptoms were also associated with lower PTSD treatment response in veterans (Simon et al. 2020). To date, many treatment programmes for refugees focus on PTSD (e.g., Kip et al. 2020), but this might disregard prolonged grief as a source of impairment, miss a source of symptom exacerbation and result in lower treatment effects.

More insights into the associations between PGD and PTSD/cPTSD in refugees are therefore needed. To investigate how symptoms of mental disorders are related to each other, the network approach to psychopathology is well suited. In network analyses, statistical relationships (represented by edges) between symptoms (represented by nodes) are estimated (Epskamp et al. 2018). Central symptoms in a network have many connections to other symptoms and may thus be especially important in the network (Borsboom and Cramer 2013).

Efforts to understand mental disorders with network analyses have increased over the last years and include studies on symptoms of post‐traumatic stress (Birkeland et al. 2020) and prolonged grief (Robinaugh et al. 2022). In many network analyses focusing on prolonged grief symptoms, *emotional pain* emerged as one of the symptoms with many connections to other symptoms (i.e., high centrality) (Maccallum et al. 2017; Maccallum and Bryant 2020; Robinaugh et al. 2014; Stelzer et al. 2020; Xu et al. 2022) and often had a particularly strong connection to *yearning* (Maccallum et al. 2017; Malgaroli et al. 2018). The symptom *feeling one has lost a part of one's self* had the highest centrality in a network of all stress‐related disorders in a representative Irish sample (Karatzias et al. 2022) and in a bereaved student sample (Bellet et al. 2018). Similarly, Malgaroli et al. (2018) identified *role confusion* as a central symptom at 3, 14 and 25 months post loss in bereaved spouses. No study has yet compared network structures of PGD symptoms between refugee and non‐refugee samples, but substantial differences have been reported for PTSD symptoms (Kangaslampi et al. 2021). Among all PTSD‐DSM‐IV symptoms, emotional numbing and concentration problems were more central in refugee samples than in non‐refugee samples, while disinterest, detachment and sleep problems were less central (Kangaslampi et al. 2021).

Focusing on the interplay between symptoms, network analysis allows a new perspective on comorbidity via identifying bridge symptoms (Cramer et al. 2010). Bridge symptoms link different symptom groups or communities within a symptom network (Cramer et al. 2010). In the network analysis of PTSD, PGD, DSO, and adjustment disorder symptoms in an Irish population‐based sample (Karatzias et al. 2022), *startle response* (PTSD) was the strongest transdiagnostically connecting symptom, and *difficulty moving on* was the prolonged grief symptom with the strongest connections to all symptoms of the other disorders. Djelantik, Robinaugh et al. (2020) examined edges between symptoms of prolonged grief, PTSD and depression in a sample of people seeking treatment for trauma‐related distress. There were strong associations among symptoms related to social disconnection and a sense of low self‐worth or purpose in life. Although this study did not include cPTSD, some of these themes resemble DSO. Investigating potential relationships among symptoms of PGD and cPTSD can further contribute to understanding comorbidities.

Insights into associations between PGD and cPTSD in refugees are limited, and the majority of existing studies has been conducted with non‐clinical samples. In the present study, we therefore combined a traditional investigation of comorbidity rates with network analysis in a sample of treatment‐seeking traumatized refugees. This can provide further insights into the mental health challenges in refugees and potentially inform diagnostic assessment as well as treatment planning and mental health policies. We recently examined PTSD_ICD‐11_ and cPTSD in this sample and found prevalences of 63.46% and 14.42%, respectively (Steil et al., forthcoming).

We first aimed to investigate the probable prevalence of PGD and its comorbidity with PTSD and cPTSD. Second, we aimed to examine central symptoms in a network of PGD, PTSD, and cPTSD symptoms and identify bridge symptoms. Given the discussion of cross‐cultural validity of mental disorders (Hinton and Lewis‐Fernández 2011) and diverging findings in refugee and non‐refugee samples (Kangaslampi et al. 2021), we focused our analyses on PGD, PTSD and cPTSD as defined in ICD‐11, because the ICD‐11 was developed with the aim of global applicability (Reed et al. 2019).

## 2 Methods

### 2.1 Procedure and Participants

Data were collected as part of a clinical trial that investigated the effectiveness of imagery rescripting for refugees with PTSD (Steil et al. 2021). The trial was conducted at four outpatient treatment centres in Germany between 2019 and 2024 and approved by the ethics committee of the German Psychological Association (transaction numbers SteilRegina2019‐10‐18‐VA and SteilRegina2020‐02‐26AM). Details about the study design and procedure are provided in the protocol paper (Steil et al. 2021).

Participants were recruited via collaborations with local service providers for refugees, healthcare providers, cultural brokers, via a project website, and (social) media. After an initial screening, interested refugees who had provided informed consent were invited to the study centres for clinical interviews and to complete self‐report questionnaires. Participants were informed about the study both verbally and in writing (via a participant information letter) and were given several days to make a decision about participation. They were informed that participation was voluntary, that they could withdraw at any time without consequences and that their data would be handled confidentially. For the present study, we used baseline data from participants who sought treatment in the trial. Independent of inclusion criteria of the clinical trial,^1^ participants were included in the present analyses if they were bereaved, had experienced a traumatic event according to Criterion A of PTSD in DSM‐5, had entered Germany as a refugee and were at least 18 years old. This resulted in a sample size of *N* = 92.

### 2.2 Measures

Trained clinician raters conducted the interviews in either German or English. If participants were not proficient in either language, a trained interpreter assisted with translation during the interview. The self‐report questionnaires were available in four languages: German, English, Arabic and Farsi. If a participant was not fluent in any of these languages or was illiterate, a trained interpreter read the items aloud and recorded the participant's responses.

Sociodemographic (i.e., gender, age, nationality, education, marital status and religion) and migration‐related characteristics (i.e., time since arrival in Germany, asylum status, living situation and employment after flight) were explored by the clinician rater during the baseline interview. Exposure to PTE was assessed with the Life Events Checklist for the Diagnostic and Statistical Manual of Mental Disorders—Interview Version (LEC‐5; Weathers et al. 2013b), which was extended to include forms of trauma related to cPTSD (e.g., torture; Lechner‐Meichsner and Steil 2021). Exposure to loss was assessed with a questionnaire on the number of deaths in the nuclear and extended family and among close friends. We also assessed the cause of death and the time since the most distressing loss.

#### 2.2.1 PGD

PGD symptoms were assessed with the Traumatic Grief Inventory‐Self Report Plus (TGI‐SR+; Lenferink et al. 2022). The TGI‐SR+ assesses PGD symptoms according to both ICD‐11 and DSM‐5‐TR and has shown good psychometric properties (Lenferink et al. 2022). Participants are asked to rate grief reactions during the past months with regard to their most distressing loss on a scale from 1 (*never*) to 5 (*always*). Total scores range between 22 and 110 with higher scores indicating higher symptom severity. Internal consistency was calculated with Mc Donald's omega using the R package psych Version 2.3.9 (Revelle 2023) and was *ω* = 0.96 in the present sample. To meet criteria for PGD_ICD‐11_, at least one symptom each of Criterion B (longing, Item 3; or preoccupation, Item 1), Criterion C (i.e., Item 2, 5, 8, 9, 10, 16, 19, 20, 21 or 22) and Criterion E (functional impairment, Item 13) had to be present (World Health Organization 2019; see also Table 1). A symptom was considered present with a score of ≥ 4 (Lenferink et al. 2022). Additionally, the loss needed to have happened more than 6 months ago.

**TABLE 1:** Diagnostic criteria for prolonged grief disorder (PGD), post‐traumatic stress disorder (PTSD) and complex PTSD (cPTSD) according to ICD‐11 and their assessment.

PGD ICD‐11		PTSD ICD‐11		cPTSD	
Symptom	Item	Symptom	Item	Symptom	Item
A: Loss of a child, partner, parent or other person close to the bereaved	Questions about the loss	A: Exposure to a stressful event or situation of exceptionally threatening or horrific nature likely to cause pervasive distress in almost anyone	LEC	A: Prolonged, repeated or multiple forms of traumatic exposure such as childhood abuse or torture	LEC
B1: Longing	TGI‐SR+ 3	B1: Re‐experiencing	CAPS 1	A: Difficulties in emotion regulation	COPISAC CO1
B2: Preoccupation	TGI‐SR+ 1	B2: Nightmares	CAPS 2	B: Negative self‐concept	COPISAC CO2
C1: Sadness	TGI‐SR+ 2	C1: Avoidance of thoughts or feelings	CAPS 6	C1: Difficulties to engage in social relationships	COPISAC CO3
C2: Guilt	TGI‐SR+ 16	C2: Avoidance of external reminders	CAPS 7	C2: Difficulties feeling emotionally close to someone	CAPS 13
C3: Anger	TGI‐SR+ 8	D1: Hypervigilance	CAPS 17		
C4: Denial	TGI‐SR+ 19	D2: Exaggerated startle response	CAPS 18		
C5: Blame	TGI‐SR+ 20	E: Impairment	CAPS 24, CAPS 25	D1: Social impairment	COPISAC CO4
C6: Difficulty accepting the loss	TGI‐SR+ 5			D2: Occupational impairment	COPISAC CO5
C7: Feeling one has lost a part of one's self	TGI‐SR+ 21	F: Duration of more than a couple of weeks	CAPS 22		
C8: An inability to experience positive mood	TGI‐SR+ 22				
C9: Emotional numbness	TGI‐SR+ 10				
C10: Difficulty engaging with social or other activities	TGI‐SR+ 9				
D: At least 6 months ago	Questions about the loss				
E: Functional impairment	TGI‐SR+ 13				
Diagnostic algorithm		Diagnostic algorithm		Diagnostic algorithm	
At least one loss of a close other AND at least one of Criterion B1 or B2 AND at least one of Criteria C1–C10 AND E are rated ≥ 4. Criterion D is fulfilled.		Having experienced at least one traumatic event AND at least one of Criterion B1 or B2 AND one of Criterion C1 or C2 AND one of Criterion D1 or D2 AND one of Criterion E1 or E2 are rated ≥ 2 AND the duration of symptoms is at least a couple of weeks.		Criteria for PTSD are met, AND Criterion A AND Criterion B AND at least one of Criterion C1 or C2 AND at least one of Criterion D1 or D2 are rated ≥ 2. Patients who meet criteria for cPTSD do not receive an additional diagnosis of PTSD.	

Abbreviations: CAPS = Clinician‐Administered PTSD Scale, COPISAC = Complex PTSD Item Set additional to the CAPS, TGI‐SR+ = Traumatic Grief Inventory‐Self Report Plus.

#### 2.2.2 PTSD_ICD‐11_ and cPTSD

We assessed PTSD_ICD‐11_ and cPTSD symptoms with the Clinician‐Administered PTSD Scale for DSM‐5 (CAPS‐5; Müller‐Engelmann et al. 2020; Weathers et al. 2013a) and the Complex PTSD Item Set additional to the CAPS (COPISAC; Lechner‐Meichsner and Steil 2021). CAPS‐5 is a structured clinical interview used to make a diagnosis of PTSD and assess the severity of 20 post‐traumatic stress symptoms in the past month according to DSM‐5 and is widely considered as the gold standard in assessing PTSD (Weathers et al. 2018). Symptoms are rated on a 5‐point Likert scale ranging from 0 (*absent*) to 4 (*extreme*). To follow ICD‐11 criteria for PTSD, CAPS items were matched to the core symptoms of PTSD as defined in the ICD‐11 guideline (see Table 1).

COPISAC is an addition to CAPS‐5 that permits the assessment of cPTSD (see Table 1). It consists of three items pertaining to DSO symptoms, and structure and scoring closely follow CAPS‐5. Total symptom severity scores for PTSD_ICD‐11_ and cPTSD were computed by summing the respective PTSD_ICD‐11_ and DSO items. Internal consistency using Mc Donald's omega was *ω* = 0.73 for PTSD_ICD‐11_ and *ω* = 0.86 for cPTSD items, respectively.

### 2.3 Data Analysis

Data analysis consisted of three parts. First, we calculated descriptive statistics to assess sample characteristics. Second, we conducted traditional analyses to investigate how many participants with probable PGD_ICD‐11_ also met diagnostic criteria for PTSD_ICD‐11_ or cPTSD and to examine associations between PGD_ICD‐11_ and PTSD_ICD‐11_/cPTSD symptom severity (Objective 1). Third, we carried out a network analysis, including network estimation and stability assessment (Objective 2). We used multiple imputation to address missing values in all steps of the analyses. All analyses were conducted using R Version 4.3.2 (R Core Team 2022). An R script demonstrating handling of missing data and traditional and network analyses is accessible on the Open Science Framework (https://osf.io/k5a68/).

Missing data ranged from 0.8% to 5.7% per scale. To address missing values, multiple imputation was conducted based on the raw item‐level data, which were later aggregated into scale scores or used to determine probable prevalence in some of the analyses. A total of 20 imputed datasets were generated using the mice package (Version 3.16.0; Van Buuren and Groothuis‐Oudshoorn 2011). The imputation procedure employed fully conditional specification with a maximum of 10 iterations, including all items relevant to the investigation of probable prevalence, comorbidity and network analysis. Predictive mean matching was used for non‐dichotomous data (with a donor pool of five observations), and logistic regression imputation was applied for dichotomous data. To prevent convergence issues due to the high number of variables relative to the number of observations, not all variables were used as predictors in every imputation model. Information regarding the time since loss was missing for some patients. However, based on biographical and other available information, we were able to determine that the loss had occurred at least 12 months prior. This information was used for multiple imputations and the estimation of probable prevalence, but not for describing sample characteristics.

#### 2.3.1 Probable Prevalence and Comorbidity

After multiple imputations, pooled probable prevalence of PGD_ICD‐11_ was assessed. We calculated the pooled numbers and proportions of participants in the total sample with a probable diagnosis of PGD_ICD‐11_. Among the participants with probable PGD_ICD‐11_, we further assessed the proportion who also met criteria for comorbid PTSD_ICD‐11_ or cPTSD according to clinical interview ratings. Pooled Pearson's product–moment correlations were used to assess associations between sum scores of symptom severity.

#### 2.3.2 Network Analysis

We estimated a regularized partial correlation network and examined the centrality and robustness of the network with the R packages networktools Version 1.5.0 (Jones 2023) and qgraph Version 1.9.8 (Epskamp et al. 2012). Multiple imputations of missing values were directly included in network estimation and bootstrapping (see below).

We estimated the network structure of the six cPTSD symptom clusters (i.e., PTSD_ICD‐11_ clusters *re‐experiencing*, *avoidance* and *sense of current threat* and DSO clusters *affective dysregulation*, *negative self‐concept* and *disturbances in relationships*) and seven PGD_ICD‐11_ symptoms. Due to the small sample size, we focused on a subset of PGD symptoms. We included the core symptoms *preoccupation* and *yearning* and accompanying symptoms that had also been included in previous network analyses and had shown high centrality or edge weights, i.e., *difficulty accepting the death*, *bitterness/anger*, *difficulty in engaging with social or other activities*, *emotional numbness* and *feeling one has lost a part of one's self* (Djelantik, Robinaugh, et al. 2020; Karatzias et al. 2022).

For four of the six cPTSD symptom clusters, a sum score of the two items assessing each cluster was used (i.e., re‐experiencing, avoidance, sense of current threat and disturbances in relationships). All other nodes were represented by a single item. The network was estimated using EBICglasso, with data modelled as ordinal (Epskamp et al. 2018). The hyperparameter was set to *γ* = 0.5. This approach to network estimation and selection was chosen because it has been shown to yield high specificity and acceptable top 5% sensitivity in settings with a low ratio of observations to nodes (Isvoranu and Epskamp 2023). This means that the strongest edges are likely to be recovered, and the edges included in the network structure can be considered genuine. To avoid recovering different network models across the 20 multiply imputed datasets, we applied a stacked imputation approach: All imputations were combined into a single dataset, and one network estimation was performed, with an adjusted sample size. This method has been shown to perform well in such contexts (Nehler and Schultze 2025).

After network estimation, we used strength and bridge strength to assess the connectedness of the nodes in the network. While strength describes the sum of all edges connected to each node, bridge strength refers to the sum of all edges between each PGD and cPTSD node, respectively. We used two theoretical communities to assess the bridge strength. The first community represented symptoms belonging to PGD, while the second community assessed symptoms belonging to cPTSD. We did not use two communities representing PTSD and DSO symptoms respectively because a diagnosis of cPTSD requires the symptoms of PTSD as well.

We used non‐parametric bootstrapping to assess the stability of the network's edge weight estimates based on 1000 bootstrap samples (Epskamp et al. 2018). The combination of bootstrapping and multiple imputations involved first performing bootstrapping, followed by generating multiple imputations (20 times) for each bootstrapped dataset. This approach has been demonstrated to produce valid confidence intervals (Schomaker and Heumann 2018). We assessed the stability of the strength and bridge strength centrality estimates using non‐parametric case‐dropping subset bootstrapping with 1000 bootstrap samples, combining multiple imputations and bootstrapping in the same way as for the edge weights. We also calculated correlation stability coefficients (CS‐coefficients; Epskamp et al. 2018). CS‐coefficients depict the highest proportion of cases that can be removed while retaining correlations of at least 0.7 with 95% probability.

## 3 Results

### 3.1 Sample Characteristics

Participants were on average 32.59 years old (SD = 11.33, range = 18–62). Most participants (*n* = 60; 65.21%) identified as male, came from Afghanistan (*n* = 29; 31.52%) or Syria (*n* = 20; 21.73%), lived in community accommodations (*n* = 44; 47.82%) or their own flat (*n* = 35; 38.04%) and had a permanent (*n* = 37; 40.21%) or temporary (*n* = 28; 30.43%) residence permit. The majority had lost a relative outside the nuclear family (*n* = 23; 25%), their father (*n* = 21; 22.82%) or a close friend (*n* = 14; 15.21%). Violent acts (*n* = 37; 40.21%) were the most frequent cause of death. Mean time since loss was *M* = 7.99 years (SD = 6.67). The most frequent index traumatic events were exposure to a war zone (*n* = 17; 18.47%), followed by sexual assault (*n* = 12; 13.04%). In line with inclusion criteria of the trial, almost all participants (*n* = 91, 98.91%) met criteria for PTSD_DSM‐5_. Sociodemographic, migration‐related and loss‐ and trauma‐related sample characteristics are depicted in Table 2.

**TABLE 2:** Sample characteristics.

	Total sample (*N* = 92)
**Sociodemographic characteristics**	
Gender, *n* (%)	
Female	29 (31.52)
Male	60 (65.21)
Non‐binary	3 (3.26)
Age in years, M (SD); range	32.59 (11.33); 18–62
Nationality, *n* (%) (1 missing)	
Afghanistan	29 (31.52)
Syria	20 (21.73)
Iraq	8 (8.69)
Iran	7 (7.60)
Nigeria	5 (5.43)
Sierra Leone	4 (4.34)
Former Yugoslavia, Guinea, Pakistan, Turkey, Eritrea, Palestine	2 (2.19)
Cameroon, Morocco, Paraguay, Egypt, Jordanian, stateless	1 (1.08)
Educational level in years of schooling, M (SD); range (1 missing)	9.05 (4.18); 0–18
Marital status, n (%)	
Single, living with family	11 (11.95)
Single, living alone	39 (42.39)
Married	20 (21.73)
Divorced	7 (7.60)
Separated	4 (4.34)
Widowed	2 (2.17)
In partnership	9 (9.78)
Religion, *n* (%) (1 one missing)	
Islam	66 (71.73)
Christianity	14 (15.21)
Non‐denominational	9 (9.78)
Other	2 (2.17)
**Migration‐related characteristics**	
Time since arrival in Germany in years, M (SD); range	5.36 (4.77); 0.17–30
Living situation, *n* (%)	
Own flat	35 (38.04)
Reception centre	3 (3.26)
Community accommodation	44 (47.82)
With family members/friends	3 (3.26)
Other	7 (7.60)
Asylum status, *n* (%)	
Temporary residence permit	28 (30.43)
Permanent residence permit	37 (40.21)
Tolerance permit	21 (22.82)
Other	6 (6.52)
Employment after flight, *n* (%)	
No	61 (66.30)
Yes	31 (33.69)
**Loss‐ and trauma‐related characteristics**	
Person who died, *n* (%) (7 missing)^a^	
Partner	1 (1.08)
Child	3 (3.26)
Mother	13 (14.13)
Father	21 (22.82)
Sibling	10 (10.86)
Other relative	23 (25.00)
Friend	14 (15.21)
Cause of death, *n* (%) (9 missing)	
Illness	33 (35.86)
Violent act	37 (40.21)
Traffic accident	4 (4.34)
Other accident	3 (3.26)
Suicide	4 (4.34)
Other	2 (2.17)
Number of losses, M, range (2 missing)	6.13, 1–39
Time since loss in years; M (SD), range (22 missing)	7.99 (6.67), 0–28
Worst traumatic event according to LEC, *n* (%)	
a: Combat or exposure to a war zone (in the military or as a civilian)	17 (18.47)
b: Captivity (e.g., being kidnapped, abducted, held hostage, prisoner of war)	3 (3.26)
c: Sudden violent death (e.g., homicide, suicide)	11 (11.95)
d: Sudden accidental death	6 (6.52)
e: Sexual assault (rape, attempted rape, made to perform any type of sexual act through force or threat of harm)	12 (13.04)
f: Other unwanted or uncomfortable sexual experience	2 (2.17)
g: Serious injury, harm or death you caused to someone else	0 (0.00)
h: Natural disaster (e.g., flood, hurricane, tornado, earthquake)	1 (1.08)
i: Fire or explosion	2 (2.17)
j: Transportation accident (e.g., car accident, boat accident, train wreck, plane crash)	1 (1.08)
k: Serious accident at work, at home or during recreational activity	0 (0.00)
l: Physical assault (e.g., being attacked, hit, slapped, kicked, beaten up)	9 (9.78)
m: Exposure to toxic substance (e.g., dangerous chemicals, radiation)	0 (0.00)
n: Assault with a weapon (e.g., being shot, stabbed, threatened with a knife, gun, bomb)	4 (4.34)
o: Life‐threatening illness or injury	3 (3.26)
p: Repeated childhood sexual abuse	2 (2.17)
q: Repeated childhood physical abuse	1 (1.08)
r: Prolonged domestic violence	3 (3.26)
s: Torture	4 (4.34)
t: Genocide campaigns	0 (0.00)
u: Being enslaved	0 (0.00)
v: Repeated medical trauma during childhood	1 (1.08)
w: Severe human suffering	1 (1.08)
x: Any other prolonged event or series of events of an extremely threatening or horrific nature from which escape was difficult or impossible	5 (5.43)
y: Any other stressful event or experience	4 (4.34)

^a^ Most distressing loss. Of the seven persons who did not indicate which loss was most distressing, one person lost a partner, two persons lost a child, one person lost their mother, three persons lost their father, four persons lost a sibling, two persons lost another relative and three persons lost a friend.

### 3.2 Probable Prevalence and Comorbidity

The pooled probable prevalence of PGD_ICD‐11_ was 28.04% (*n* = 26). Similar to our previous study (Steil et al., forthcoming), pooled prevalence was 64.13% (*n* = 59) for PTSD_ICD‐11_ and 15.98% (*n* = 15) for cPTSD. Among participants with a probable diagnosis of PGD_ICD‐11_, 19.23% (*n* = 5) also met criteria for cPTSD, and 65.38% (*n* = 17) also met criteria for PTSD_ICD‐11_. In the total sample, 18.47% had comorbid PGD_ICD‐11_ and PTSD_ICD‐11_, and 5.43% had comorbid PGD_ICD‐11_ and cPTSD.^2^ Pooled means and standard deviations of symptom severity for PGD_ICD‐11_, PTSD_ICD‐11_ and cPTSD are depicted in Table 3. All correlations among sum scores were significant (*p* < 0.001; see Table 3). PGD_ICD‐11_ symptom severity had low correlations with symptom levels of PTSD_ICD‐11_ (*ρ* = 0.23), cPTSD (*ρ* = 0.24) and DSO (*ρ* = 0.16).

**TABLE 3:** Pooled means, standard deviations and correlations of TGI‐SR+, CAPS and COPISAC.

	M (SD)	1	2	3
1. PGD	65.34 (23.83)			
2. PTSD_ICD‐11_	11.68 (4.24)	0.23***		
3. DSO	5.72 (4.09)	0.16***	0.35***	
4. cPTSD	17.41 (6.84)	0.24***	0.82***	0.81***

Abbreviations: PGD = prolonged grief disorder, PTSD_ICD‐11_ = post‐traumatic stress disorder according to ICD‐11, DSO = Disorders of self‐organization, cPTSD = complex post‐traumatic stress disorder.

*p < 0.05, **p < 0.01, ***p < 0.001.

### 3.3 Network Analysis

**FIGURE 1:**
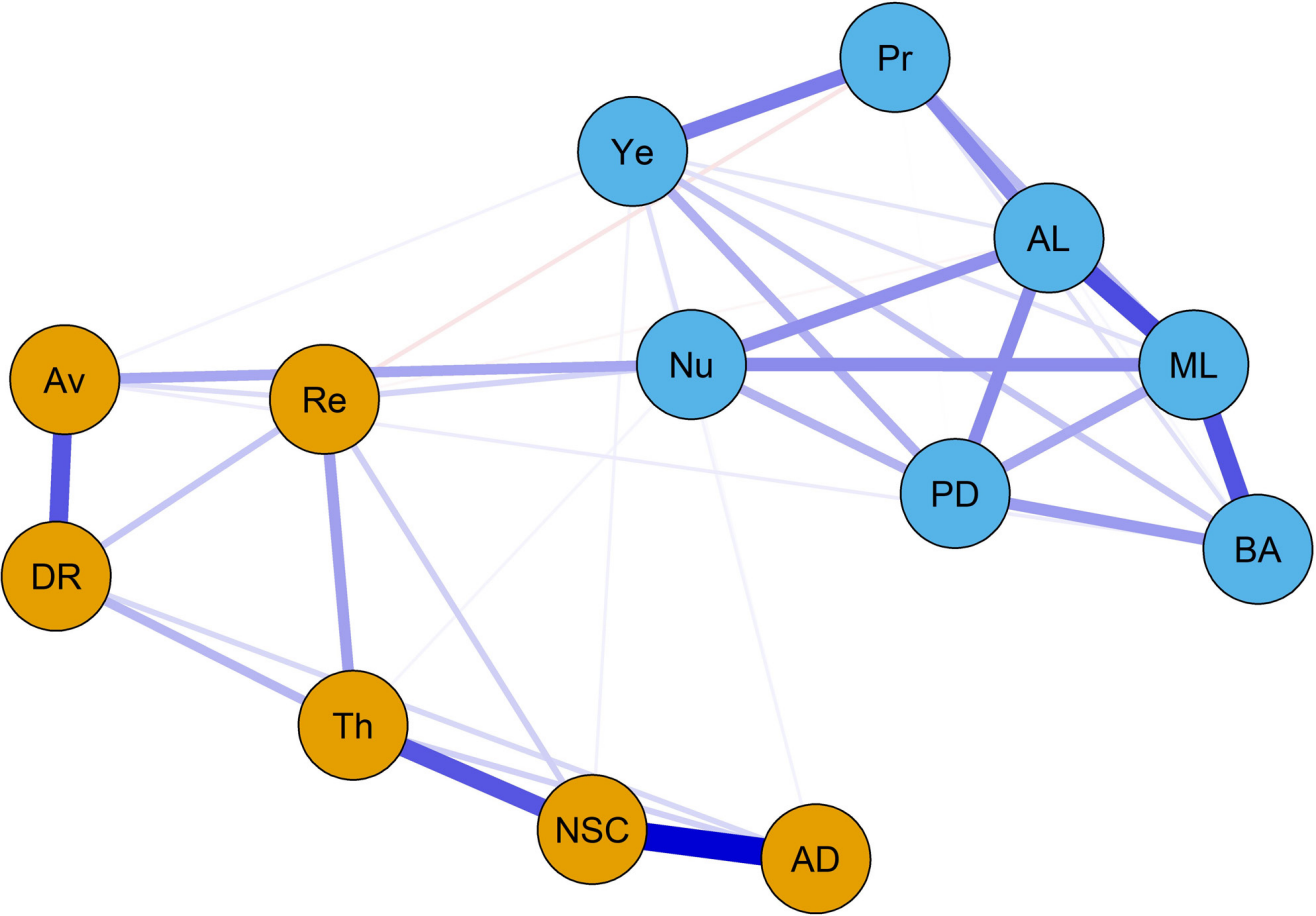
Network visualization. *Note:* Regularized partial‐correlation network (tuning parameter *γ* = 0.5) of the seven PGD symptoms (blue nodes) and six cPTSD symptom clusters (orange nodes). The strength of the association is represented by edge thickness. Positive, regularized partial correlations are depicted by blue edges, and negative, regularized partial correlations are depicted by red edges. ad = affective dysregulation, AL = difficulties accepting the loss, Av = avoidance, BA = bitterness or anger, DR = disturbances in relationships, ML = difficulties in engaging with social or other activities, NSC = negative self‐concept, Nu = emotional numbness, PD = feeling one has lost a part of one's self, Pr = preoccupation, Re = re‐experiencing, Th = sense of current threat, Ye = yearning.

**FIGURE 2:**
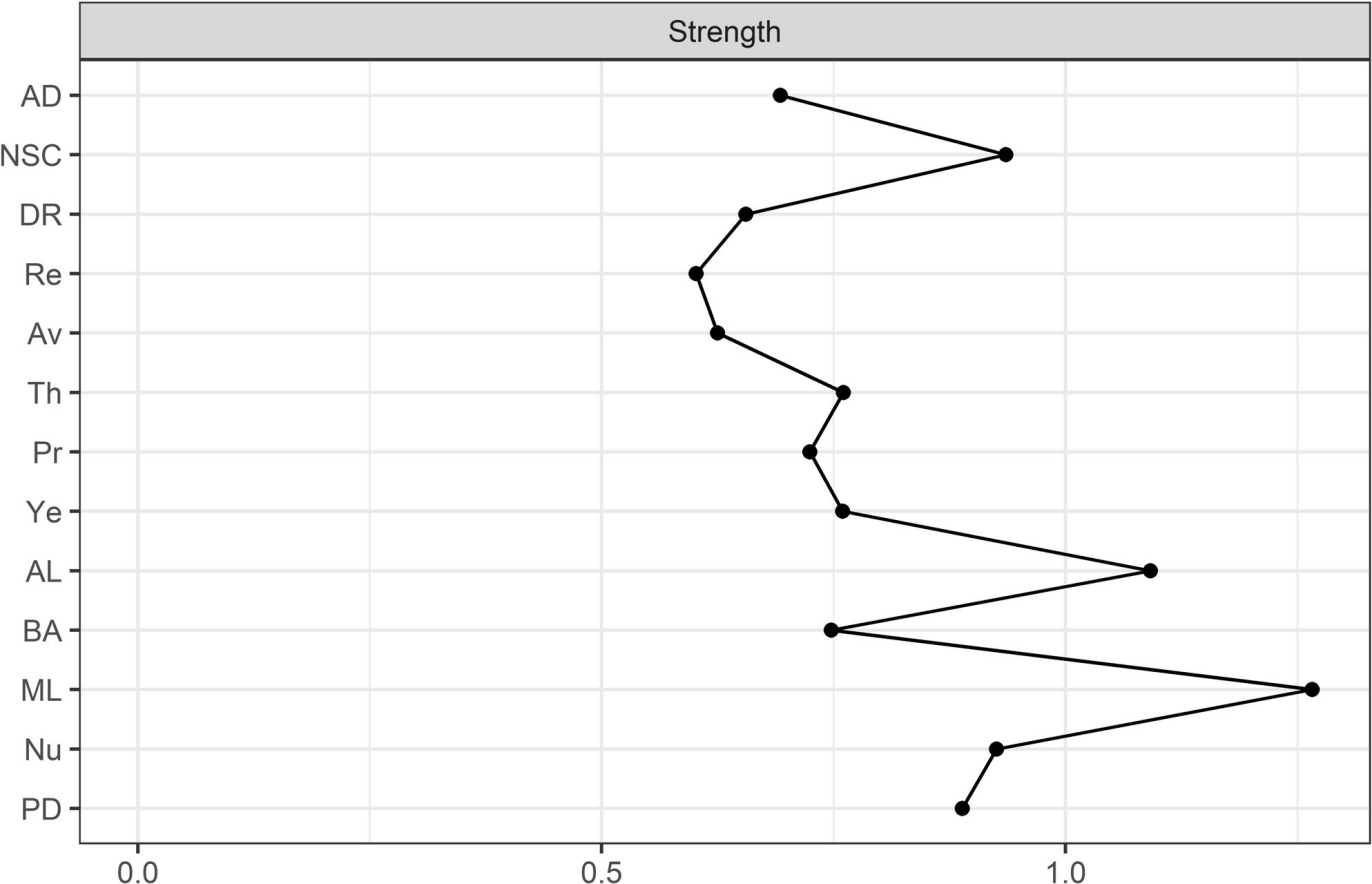
Centrality plot for strength centrality results for seven PGD symptoms and six cPTSD symptom clusters. ad = affective dysregulation, AL = difficulties accepting the loss, Av = avoidance, BA = bitterness or anger, DR = disturbances in relationships, ML = difficulties in engaging with social or other activities, NSC = negative self‐concept, PD = feeling one has lost a part of one's self, Pr = preoccupation, Re = re‐experiencing, Th = sense of current threat, Ye = yearning, Nu = emotional numbness.

**FIGURE 3:**
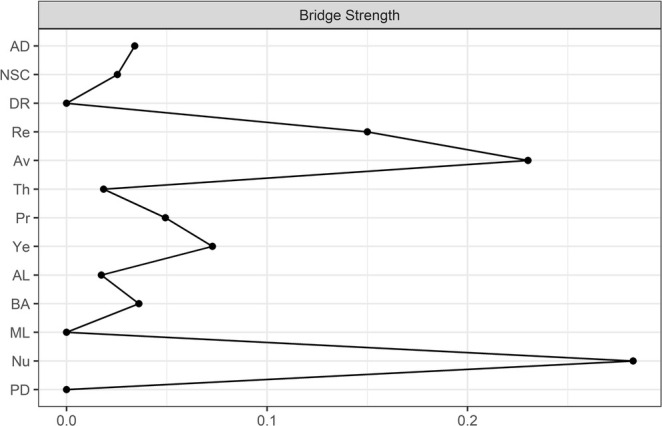
Centrality plot representing bridge centrality results for seven PGD symptoms and six cPTSD symptom clusters. ad = affective dysregulation, AL = difficulties accepting the loss, Av = avoidance, BA = bitterness or anger, DR = disturbances in relationships, ML = difficulties in engaging with social or other activities, NSC = negative self‐concept, Nu = emotional numbness, PD = feeling one has lost a part of one's self, Pr = Preoccupation, Re = re‐experiencing, Th = sense of current threat, Ye = yearning.

The visualization of the estimated network is depicted in Figure 1. We used the colorblind theme in *qgraph* (Epskamp et al. 2012). The layout was generated automatically using the Fruchterman–Reingold (1991) algorithm. The size of the non‐zero edge weights ranged from −0.04 (*preoccupation*–*re‐experiencing*) to 0.49 (*affective dysregulation*–*negative self‐concept*). The mean edge weight was 0.06, and the density was 0.5. The next highest edge weight was between the PGD symptoms *difficulties accepting the loss* and *difficulty in engaging with social or other activities* (0.34). The results of the centrality analyses are depicted in Figures 2 and 3. The two most central symptoms belonged to PGD and were *difficulty in engaging with social or other activities* (1.26) and *difficulties accepting the loss* (1.09). The two most central symptoms belonging to cPTSD were the DSO symptom *negative self‐concept* (0.93) and the PTSD symptom *sense of current threat* (0.76). The strongest PGD bridge symptom was *emotional numbness* (0.28) while the strongest cPTSD bridge symptom was *avoidance* (0.23).

The confidence intervals for the edge weights overlapped largely, which indicates that most edge weights cannot be differentiated stably from each other. However, the confidence intervals of the two highest edge weights (i.e., *affective dysregulation*–*negative self‐concept* and *difficulties accepting the loss*–*difficulty in engaging with social or other activities*) indicate that those edge weights are significantly larger than the lower edge weights. Non‐parametric bootstrapping results are depicted in Figure S4.

The strength and bridge strength stability coefficients indicated that 45.65% and 22.83% of the data could be dropped to retain a correlation of 0.7 with 95% certainty. The plot of the case‐dropping subset bootstrapping is depicted in Figure S5.

## 4 Discussion

The aim of the present study was to explore symptoms of PGD and their relation to PTSD and cPTSD in a sample of treatment‐seeking traumatized refugees. We determined the probable prevalence of PGD and rates of comorbidity with PTSD and cPTSD and used network analysis to identify central symptoms as well as bridge symptoms between disorders. Combining the symptom‐level approach of network analysis with traditional analyses of comorbidity provided novel insights into the comorbidity of PGD in a clinical sample. Given the heterogeneity of our sample regarding the cultural background, we focused our analyses on ICD‐11 criteria because they were developed with the goal of global applicability.

In the present sample, 28.04% of participants met criteria for probable PGD. This rate falls within the range reported for prolonged grief in treatment‐seeking refugees (Lechner‐Meichsner et al. 2024) and also in studies with refugees that applied ICD‐11 criteria (15.1% to 36.8%; Bryant, Bawaneh, et al. 2021; Bryant, Edwards, et al. 2021; Bryant et al. 2019). It is notably higher than the probable prevalence reported by Bryant, Bawaneh, et al. (2021) who assessed Syrian refugees for eligibility for a psychological intervention but slightly lower than in a population‐based study with refugees in Australia (Bryant, Edwards, et al. 2021). Importantly, we did not study a representative sample but refugees seeking treatment for PTSD. In addition, for many participants, the loss did not involve a first‐degree relative, and only 40.21% were traumatically bereaved. As violent loss and a close relationship to the deceased are risk factors for prolonged grief (Buur et al. 2024), higher prevalences can be expected in samples with these characteristics.

Most participants with probable PGD also met criteria for PTSD or cPTSD, with comorbid PTSD being more frequent than comorbid cPTSD. Given that participants were seeking treatment for PTSD, this high rate of comorbidity is not surprising. Nearly a quarter of all participants met criteria for probable PGD in connection with PTSD or cPTSD. In a study with refugees who resettled in Germany, 44% met criteria for prolonged grief and PTSD according to DSM‐5 (Comtesse and Rosner 2019). The slightly lower proportion in our study can be attributed to the stricter PTSD criteria in ICD‐11 compared to DSM‐5, which often leads to lower prevalence estimates (Heeke et al. 2020). For a discussion of the overlap between ICD and DSM criteria for PTSD, see First et al. (2021). Studies that identified subgroups with similar symptom profiles among refugees or internally displaced people also consistently found that between 10% and 36.7% of the sample displayed symptoms of both prolonged grief and PTSD next to a group that was characterized by PGD symptoms alone (Comtesse et al. 2024; Jann et al. 2024; Nickerson et al. 2014; Tay et al. 2019). A higher co‐occurrence between prolonged grief and post‐traumatic stress has been reported in the literature (Komischke‐Konnerup et al. 2021). The lower rate of co‐occurrence in our study likely reflects the fact that we did not focus on bereavement‐related PTSD but included participants who had experienced diverse types of traumatic events. The present study provides additional insights into PGD and cPTSD by investigating correlations between symptom levels. The low correlations between symptom levels resemble those reported by Silove et al. (2017) who also studied a sample exposed to different traumatic events.

Network analysis was used to investigate the relationships between symptoms of PGD and cPTSD to provide insights into the interplay of symptoms. The symptom with the strongest link to all other symptoms in the network was the PGD symptom *difficulty in engaging with social or other activities*. This indicates that this symptom either activates many other symptoms or is activated by many other symptoms. *Difficulties in engaging with social or other activities* also had high centrality in an Irish sample (Karatzias et al. 2022), an African sample (Robinson et al. 2024) and an international sample (Killikelly et al. 2023), but other studies also reported low centrality (Maccallum and Bryant 2020). From a theoretical perspective, patterns of inactivity and withdrawal from activities that were enjoyed before the loss have been termed depressive avoidance (Boelen et al. 2006). Avoidance has been proposed as a core process in the development and maintenance of PGD symptoms (Boelen et al. 2006) and is supported by robust empirical evidence (e.g., Boelen 2021; Boelen and van den Bout 2010; Eisma and Stroebe 2021). Although our analysis was cross‐sectional, the importance of *difficulty in engaging with social or other activities* in the network may add to the empirical evidence for this relationship. The high centrality in our sample may also partly stem from refugees' limited resources and opportunities due to postmigration difficulties, which can further hinder their ability to pursue activities.

The second most central symptom in the network was *difficulties accepting the loss* (PGD), which also resembles findings obtained in some earlier studies (Killikelly et al. 2023; Maccallum et al. 2017) but differs from others (Maccallum and Bryant 2020; Xu et al. 2022). Importantly, difficulties accepting the loss have been related to a poor integration of the loss into the autobiographical memory, another core process that maintains PGD symptoms (Boelen et al. 2006). Among the PGD symptoms, we also found the highest edge weight between *difficulties accepting the loss* and *difficulty in engaging with social or other activities*. Although our cross‐sectional analysis precludes conclusions about the direction of this association, it seems likely that the lack of acceptance of the loss and the inherent focus on the deceased and the past directly impede the more future‐oriented engagement in other relationships and activities.

Results regarding symptom centrality therefore align with existing empirical findings and theoretical conceptualizations of PGD. However, in contrast to other network studies of prolonged grief symptoms (Maccallum et al. 2017; Malgaroli et al. 2018), *yearning* and *feeling one has lost a part of one's self* were less central. Yearning/longing also showed lower centrality in other non‐Western samples (Killikelly et al. 2023; Robinson et al. 2024; Xu et al. 2022), which might hint at a possible cultural difference. Discrepancies regarding central prolonged grief symptoms could also stem from other sources. First, different risk factors might have an influence on centrality. Second, the number and content of included nodes apart from prolonged grief symptoms can influence edge weights and centrality. For example, Maccallum and Bryant (2020) included prolonged grief symptoms and different facets of quality of life in their network. Different nodes may be influential when quality of life, rather than cPTSD symptoms, is included in a network. Third, the wording of items used to assess symptoms might influence the importance in the network, especially in one‐word ICD‐11 PGD symptoms that have been criticized as ambiguous (Eisma et al. 2020).

Regarding cPTSD, the high centrality of *sense of negative self‐concept* is in line with results obtained by Karatzias et al. (2022) in a representative Irish sample, adding to the importance of that symptom cluster across different samples.

To better understand the comorbidity between PGD and cPTSD, we identified bridge symptoms. The PGD symptom *emotional numbness* had the highest bridge strength, i.e., seemed most strongly related to all cPTSD symptoms. This is not surprising given that (a) affect dysregulation in cPTSD can also take the form of emotional numbing or hypoarousal (World Health Organization 2019) and (b) PTSD has been associated with emotional numbness and lack of emotional response in general (Duek et al. 2023). Interestingly, emotional numbness was also identified as a link between symptoms of PGD and depression (Robinaugh et al. 2014), suggesting it is a transdiagnostic symptom. *Avoidance* had the highest bridge strength of all cPTSD symptoms. This result differs from the network analysis in Chinese bereaved parents where avoidance had low bridge strength (Xu et al. 2022) but is similar to results reported by Djelantik, Robinaugh et al. (2020), who found a strong intercommunity edge between avoidance in PTSD and prolonged grief in a sample that also consisted of treatment‐seeking patients after trauma. Avoidance is not part of the ICD‐11 criteria for PGD, but patients with PGD often engage in avoidance of reminders of the loss, which can directly influence PGD symptoms (Boelen et al. 2006). While the focus of avoidance differs between PTSD and PGD, the link between PTSD‐related avoidance and PGD symptoms in our sample may thus reflect a general tendency to engage in avoidance, which can activate other PGD symptoms and contribute to comorbidity.

### 4.1 Limitations

The study has several limitations that should be kept in mind when interpreting our findings. Most importantly, the sample size is small for network analysis, which is reflected in the low stability values. While strength centrality exceeded the minimum recommended threshold of 25% for the case‐dropping bootstrap (as suggested by Epskamp et al. 2018), it did not reach the ideal cutoff of 50%. Bridge strength fell below both thresholds. Therefore, the results concerning centrality indices should be interpreted with caution and require replication. Furthermore, while the included edges can be considered genuine and are likely among the strongest in the true underlying network structure—due to the chosen approach to network analysis—this does not imply that all edges not present in the network are truly absent in the population. Nevertheless, sample sizes similar to ours are not uncommon in studies involving refugee populations, including network analyses (Schiess‐Jokanovic et al. 2022), particularly when clinical ratings are collected with the assistance of interpreters. Moreover, the present study contributes meaningfully to the literature by extending previous research, which has primarily relied on self‐report measures (Karatzias et al. 2022; Lechner‐Meichsner et al. 2024) through the use of clinician‐administered assessments. The small sample size also precluded including all PGD_ICD‐11_ symptoms as nodes, which is why we opted for a selection guided by previous research. The type and number of symptoms included in network analyses of prolonged grief to date vary greatly, which is likely a result of the development of diagnostic criteria and corresponding instruments over the last years. We selected items based on their inclusion in previous studies with similar aims and samples but cannot rule out that we missed influential symptoms. Therefore, future research should investigate networks of PGD symptoms more systematically, using the same measures to assess symptoms, include the same nodes and then vary populations and other symptoms in a second step to facilitate comparability.

Due to the cross‐sectional nature of our analyses, no conclusions about causal influences between symptoms are possible. In order to understand the direction of symptom activation in the system, longitudinal investigations are needed. It seems especially interesting to extend the findings on the influence of PGD symptoms on PTSD to cPTSD and unveil how DSO and PGD symptoms relate to each other over time.

Although the CAPS‐5 and the TGI‐SR+ have been used with refugee samples before, the measures have not been culturally adapted for the use with the groups included in our study. The interview setting and the presence of interpreters allowed us to give explanations when participants did not understand certain symptoms or item formulations. However, our assessment may have missed culture‐specific reactions (e.g., somatic symptoms; Killikelly et al. 2021) that are relevant for a true prevalence estimation.

Finally, the results cannot be generalized to all refugees. We conducted our analyses in a sample seeking treatment for PTSD in a clinical trial, and participants were primarily screened for eligibility in the trial. While our study yields important insights into a treatment‐seeking group with high symptom severity, further investigations in representative refugee samples are needed.

### 4.2 Implications

Keeping the limitations of our study in mind, the present results add to the evidence for PGD as an important mental health problem in refugees. They underline the importance of assessing bereavement alongside trauma history and the need to screen for PGD in bereaved refugees, even when they seek treatment for other complaints, and offer grief‐focused treatments. While treatment programmes for refugees often address PTSD (e.g., Kip et al. 2020), future research needs to widen their focus to also include PGD. To date, some culturally sensitive grief‐focused treatment programmes exist (Aeschlimann et al. 2024), and a treatment for traumatic loss that combines interventions for PGD and PTSD and allows us to incorporate cultural aspects has shown promising effects (Djelantik, de Heus, et al. 2020; Smid et al. 2015).

It has been suggested that central symptoms in network analyses are candidates for intervention targets (McNally 2016). This claim is debated because it is unclear whether centrality also implies influence (Bringmann et al. 2019) and because cross‐sectional data cannot differentiate between within‐person and between‐person effects (Hamaker 2012) and interventions are person‐focused. Cross‐sectional research can still help to explore the structure of symptoms (Spector 2019) and provide initial pointers, with strength being the most reliable centrality index (Bringmann et al. 2019). Following McNally's (2016) argument, our results suggest a focus on (re‐)engaging in activities and accepting the loss. Exposure can facilitate the integration of the loss into the autobiographical memory and thereby also acceptance of the loss (Boelen et al. 2006), and it is a central component in most efficacious treatments for prolonged grief symptoms (Komischke‐Konnerup et al. 2024). When applied as an intervention for prolonged grief symptoms, exposure is focused on internal and external loss‐related stimuli (e.g., distressing memories and painful aspects of the loss, as well as places or activities that are associated with the deceased and avoided).

A focus on engaging in social and other activities might be especially important for refugees who are often isolated or lack social support due to migration (Belau et al. 2021). Many treatments for prolonged grief symptoms include behavioural activation to counter inactivity and withdrawal (Komischke‐Konnerup et al. 2024), and positive effects of this intervention have been shown (e.g., Eisma et al. 2015). Some treatments also focus explicitly on increasing social support (Shear and Gribbin Bloom 2017).

### 4.3 Conclusion

Network analysis has allowed important insights into associations between symptoms of PGD and cPTSD and contributed to our understanding of the comorbidity between PGD and PTSD/cPTSD in a sample of traumatized refugees. The high probable prevalence of PGD emphasizes the need to include PGD in the assessment of treatment‐seeking refugees and offer grief‐focused culturally sensitive treatments.

## Author Contributions

**Franziska Lechner‐Meichsner:** conceptualization, data curation, methodology, project administration, writing – original draft. **Mirjam Sophie Rueger:** conceptualization, data curation, formal analysis, methodology, project administration, writing – original draft. **Kai Jannik Nehler:** data curation, formal analysis, methodology, visualization, writing – original draft. **Thomas Ehring:** funding acquisition, supervision, writing – review and editing. **Hannah Preiss (nee Schumm):** investigation. **Nexhmedin Morina:** funding acquisition, supervision, writing – review and editing. **Dana Churbaji:** investigation. **Ricarda Mewes:** writing – review and editing. **Julia Giesebrecht:** investigation. **Cornelia Weise:** writing – review and editing. **Regina Steil:** conceptualization, data curation, funding acquisition, methodology, supervision, writing – review and editing.

## Conflicts of Interest

The authors declare no conflicts of interest.

## Supporting information


**Figure S4.** Nonparametric bootstrapping results with 1000 samples for the network of seven PGD symptoms and six cPTSD symptom clusters.

**Figure S5.** Stability of strength and bridge strength centrality estimates for the network of seven PGD symptoms and six cPTSD symptom clusters.

**Data S1.** Supporting information.

## Data Availability

Due to the sensitive nature of the questions asked in this study, study participants were assured raw data would remain confidential and would not be shared. However, we created a dummy dataset that can be accessed alongside the R code on the Open Science Framework (https://osf.io/k5a68/).

## Endnotes

^1^
Participants were eligible for the trial if they had a primary diagnosis of PTSD according to DSM‐5, had entered Germany as a refugee, were between 18 and 65 years old, were able to communicate with the therapist with or without the help of an interpreter, were motivated to undergo trauma‐focused treatment, had health insurance that covers psychological treatment costs and were able to stay in the location of the study centre for at least another 6 months. Exclusion criteria were a lifetime diagnosis of psychosis, bipolar disorder or substance dependence, acute suicide risk or risk of harm of others and start of new medication for mental health problems within 1 month prior to the study.

^2^
In response to reviewer feedback, we explored the impact of different diagnostic thresholds on PGD prevalence and comorbidity estimates. Applying the ICD‐11 definition (≥ 1 accompanying symptom) yielded a PGD prevalence of 28.04%. This estimate decreased only modestly when using up to seven accompanying Criterion C symptoms (probable prevalence 23.21%) for diagnosis and dropped more substantially at ≥ 8 (probable prevalence 16.90%) and ≥ 10 (probable prevalence 7.34%) Criterion C symptoms. Comorbidity rates with cPTSD remained relatively stable across thresholds (5.43% at one to eight symptoms), whereas comorbidity with PTSD declined more notably (from 18.47% at one symptom to 4.34% at 10 symptoms).
