# Supplementary figures and images for: Prolonged Grief in Refugees Seeking Treatment for PTSD: Comorbidity with Post‐Traumatic Stress Symptoms and Network Structure
Source: Clin Psychol Psychother. 2025 Jun 21;32(3):e70097. doi: 10.1002/cpp.70097 (PMC12181821; doi:10.1002/cpp.70097)

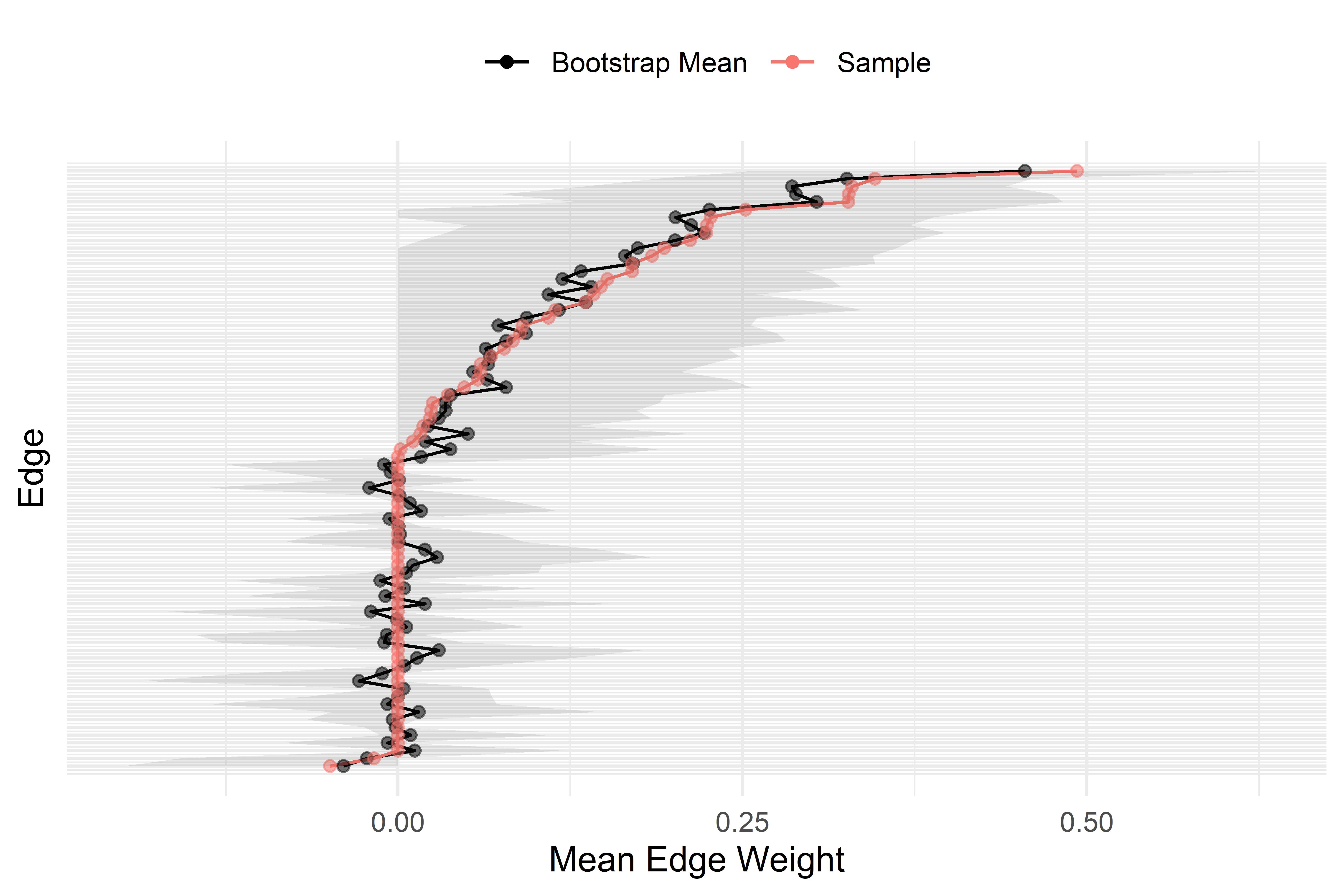

Supplement: Supplementary file 1 — Figure S4. Nonparametric bootstrapping results with 1000 samples for the network of seven PGD symptoms and six cPTSD symptom clusters. [file CPP-32-e70097-s003.jpeg]

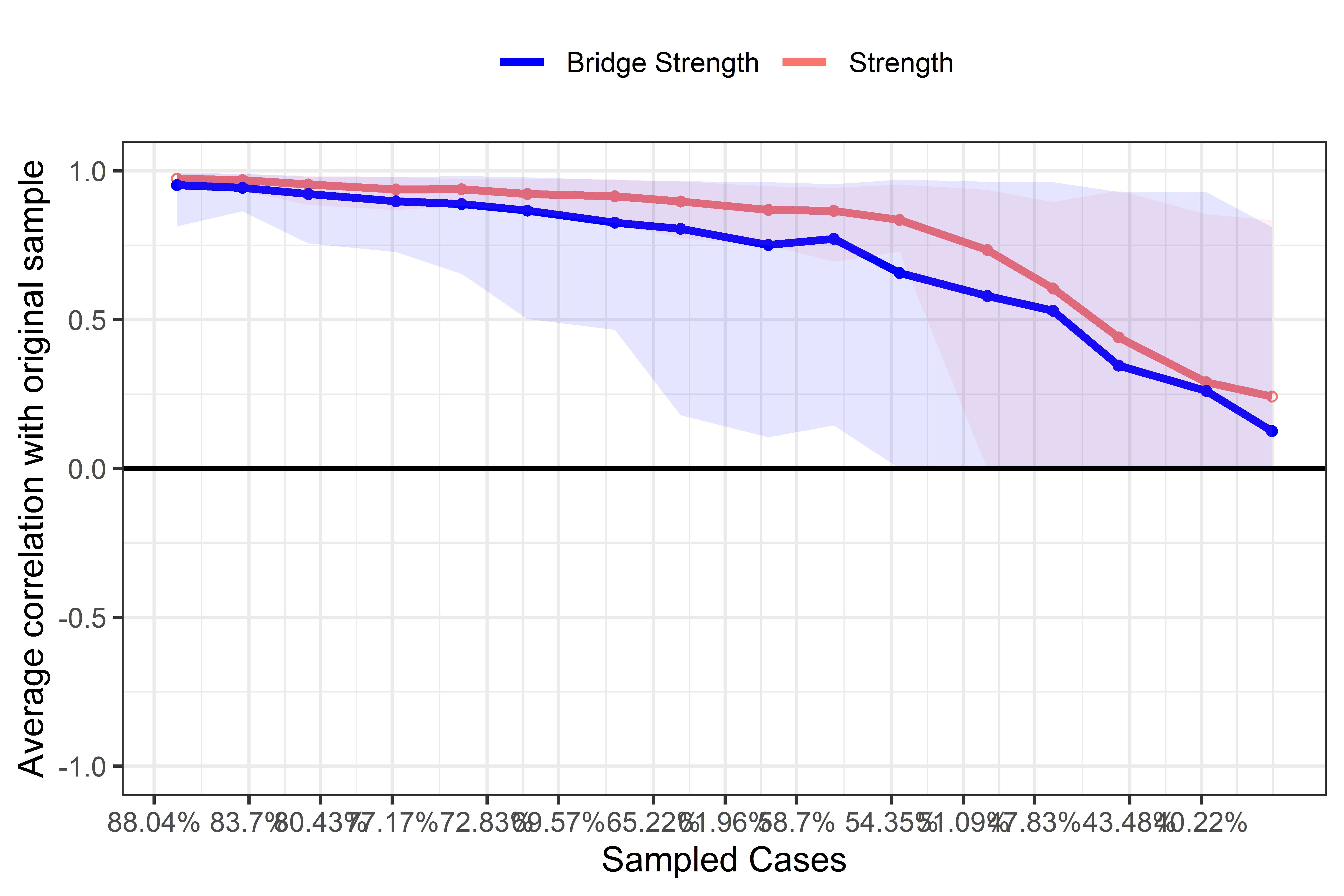

Supplement: Supplementary file 2 — Figure S5. Stability of strength and bridge strength centrality estimates for the network of seven PGD symptoms and six cPTSD symptom clusters. [file CPP-32-e70097-s002.jpeg]
