# Supplementary material for: Prolonged Grief in Refugees Seeking Treatment for PTSD: Comorbidity with Post‐Traumatic Stress Symptoms and Network Structure
Source: Clin Psychol Psychother. 2025 Jun 21;32(3):e70097. doi: 10.1002/cpp.70097 (PMC12181821; doi:10.1002/cpp.70097)
Supplement: Supplementary file 3 — Data S1. Supporting information. [file CPP-32-e70097-s001.docx]

Supplementary Material

| 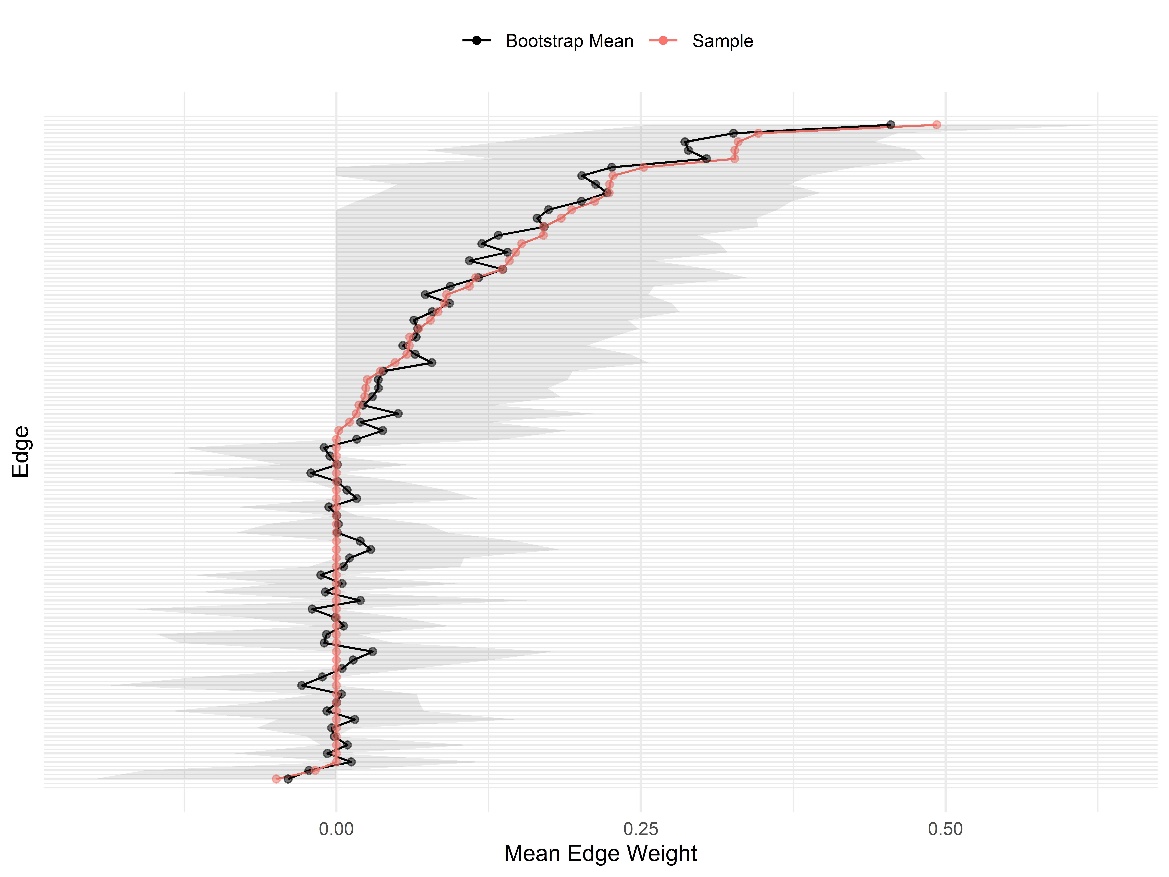 |
| --- |
| *Figure 4.* Nonparametric bootstrapping results with 1000 samples for the network of seven PGD-symptoms and six cPTSD symptom clusters. |

| 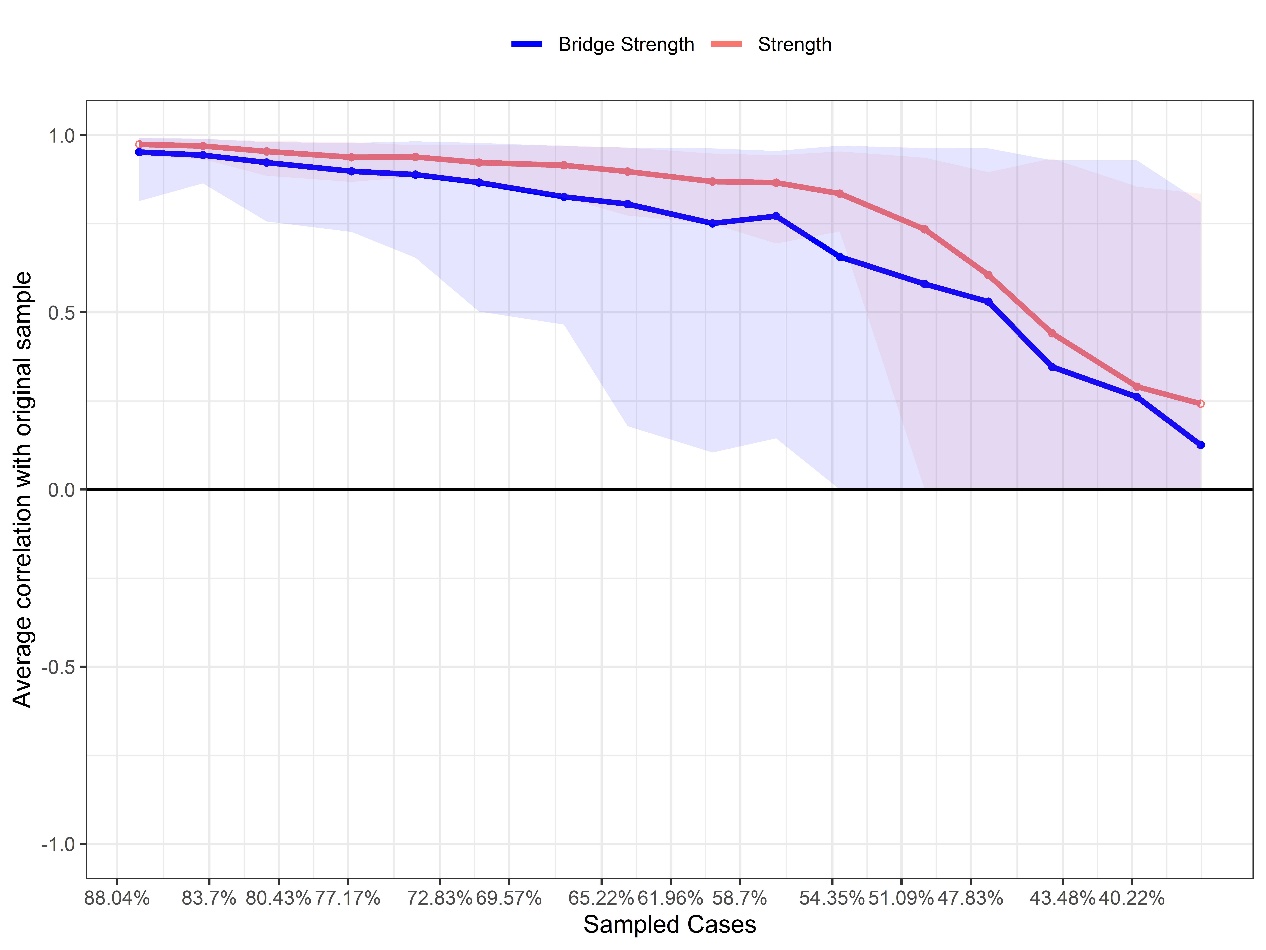 |
| --- |
| Figure 5. Stability of strength and bridge strength centrality estimates for the network of seven PGD-symptoms and six cPTSD symptom clusters |
